# Supplementary material for: Estimating the uncertain effect of the COVID pandemic on drug overdoses
Source: PLoS One. 2023 Aug 10;18(8):e0281227. doi: 10.1371/journal.pone.0281227 (PMC10414597; doi:10.1371/journal.pone.0281227)
Supplement: S1 Appendix — (DOCX) [file pone.0281227.s001.docx]

**Supplementary Appendix for:**

**The Uncertain Effect of the COVID Pandemic on Drug Overdoses**

Ali Moghtaderi, PhD, MBA^1^, Mark S. Zocchi, MPH^2^, Jesse M. Pines, MD^3^, Arvind Venkat, MD^3^, Bernard Black, JD, MA^4^

^1^ Milken Institute School of Public Health, George Washington University, Washington, DC

^2^ Heller School for Social Policy and Management, Brandeis University, Waltham, MA

^3^ US Acute Care Solutions, Canton, OH and Department of Emergency Medicine, Allegheny Health Network, Pittsburgh, PA

^4^ Pritzker School of Law and Kellogg School of Management, Northwestern University, Evanston, IL

**Literature Ascribing Rising Overdose Deaths and ED Visits to the COVID Pandemic**

*Overdose deaths.* Appa et al. (2021) [1] compared overdose deaths, averaged over July 2019-mid-March 2020 to deaths from mid-March through November 2020 in San Francisco. They attribute the observed increase in overdose deaths to the pandemic, but do not model pre-pandemic trends. Yet their own data show rising mortality in the pre-pandemic period, from 241 in 2018 to 436 in 2019, including a rise from 174 in the first half of 2019 to 262 in the second half. (Supplemental Appendix Table-1). San Francisco was also among the cities that were impacted by a surge in supply of illicitly manufactured fentanyl that began in mid-2019.[2]

Mason et al. (2021) [3] studied trends in opioid-overdose deaths in Cook County, Illinois, from 2018 through mid-2020. They showed that the opioid-related mortality peaked during the time that a stay-at-home order was in place (March 21-June 5, 2020), and declined after the order was lifted. However, opioid overdose deaths in Cook County began rising in late 2019, and remained well above the late 2019 level after the stay-at-home order was lifted. The authors also report that the percent of fentanyl-related deaths increased steadily over their study period. These findings suggest that the pandemic was at most a partial explanation for higher overdose deaths in the early pandemic period.

Currie et al (2021) [4] report trends in overdose deaths, divided into fentanyl-related, heroin, all other opioids, and other drugs, from 2018 to October 2020 in Ohio. They report a rise in fentanyl-related overdoses in April and May 2020, which recedes to roughly pre-pandemic levels by August 2020; but little change for other drugs. They do not explicitly model pre-pandemic trends but their Figure 1 shows evidence of a short-lived spike in fentanyl-related deaths early in the pandemic.

Kelley et al (2021) [5] found higher opioid-related deaths in February-April 2020 versus the same months in 2019, in Los Angeles County, and ascribe higher 2020 deaths to the pandemic, even though their own data (their Figure 1) shows that the year-over year increase was larger in February than in March or April without consideration of pre-pandemic trends in increasing trajectory death before the COVID-19 pandemic.

Faust et al. (2021) [6] study the period from March-August 2020. This is the only prior study that models counterfactual overdose mortality. They use an autoregressive model, which produces unreasonable predictions. Despite the increase in opioid overdose deaths that began in mid-2019, their model predicts overdose deaths that fall during their sample period, and are lower than observed deaths.

In short, none of the prior studies provides convincing evidence that the pandemic, as opposed to continuation of pre-pandemic trends, was a primary cause of the 2020 rise in overdose deaths. Four (all but Faust) report overdose deaths for February 2020 versus February 2019; of these three (all but Currie) find a sharp rise in overdose deaths in February 2020, prior to the pandemic. Currie et. al (2021) report evidence for a time-limited spike in fentanyl deaths, but only for a single state.

*ED Visits.* Soares et. al (2021) [7] studied opioid ED visits, both raw counts and as a fraction of all ED visits, among 6 health systems in 6 states. They compared ED counts and rates during 2018-2019 to counts and rates in 2020, and report higher overdose visit counts in 2020, and declining visits from other causes. The study simply compares 2020 to the 2018-2019 average. It reports 3,020 opioid-related ED visits in 2018, 3,285 in 2019 (9% increase over 2018), and 3,486 in 2020 (6% increase over 2019). We view these results as consistent with a general rise in overdose-related ED visits of their study period, with no evidence that trends accelerated during the pandemic period.

Holland et. al (2021) [8] studied overdose-related ED visits over December 2018-October 10, 2020, relying on internal CDC data covering 3,500 EDs in 48 states. They compared counts and rates for overdose-related ED visits before and after the pandemic onset without a counterfactual. They report higher pandemic period ED visits, but their weekly counts (their Figure 1) show no change in trends at the pandemic onset.

Ochalek et. al (2020) [9] compared average non-fatal opioid overdose visits to a single Virginia ED over March- June 2019 to a similar time period in 2020, and found substantially more visits in 2020. This study did not examine trends between the two periods, which might partially or fully explain higher 2020 ED visit counts.

Slavova et. al (2020) [10] report changes in daily emergency medical services (EMS) calls in Kentucky during a narrow period around the March declaration of a pandemic emergency (26 days before vs. 26 days after the emergency declaration). They do not assess pre-pandemic trends. This time period is too short to draw solid conclusions, especially since the pandemic could have affected the proportion of ED visits that involve EMS calls.

Rodda et. al (2020) [11] studied opioid-related deaths in San Francisco and opioid-related ED visits to two San Francisco EDs from January 1 through April 18, 2020, a total of 176 visits, without accounting for pre-existing trends. The short study period and small number of observations make it difficult to draw conclusions from this paper. Unlike Appa et. al (2021)^1^ who studied San Francisco over a longer time period, they did not report a pandemic-period increase in opioid mortality.

Our own prior study [12] covers January 2019 through July 2020, compares pandemic to pre-pandemic overdose visit rates, and finds generally higher visit rates in 2020, but similar 2020/2019 visit ratios during the pandemic period versus earlier in 2020.

In sum, the available evidence on overdose-related ED visit rates supports rising visit rates during both the pre-pandemic and pandemic periods but does not provide convincing evidence for a pandemic-related change in visit trends.

**List of states in the national non-fatal ED visits database**

Below is list of states included in the non-fatal ED visit database: Alabama, Alaska, Arizona, Arkansas, Colorado, Connecticut, Delaware, District of Columbia, Florida, Georgia, Idaho, Illinois, Indiana, Kansas, Kentucky, Louisiana, Maine, Maryland, Massachusetts, Michigan, Mississippi, Missouri, Montana, Nevada, New Hampshire, New Jersey, New Mexico, New York, North Carolina, Ohio, Oregon, Pennsylvania, Rhode Island, South Carolina, South Dakota, Tennessee, Utah, Vermont, Virginia, Washington, West Virginia, and Wisconsin.

**Breakdown of EDs by states in the staffing Company Data**

The breakdown of 181 EDs by states is reported in Appendix Table 2.

**Placebo pandemic results**

In Appendix Figure 1, we report results from a placebo analysis, in which we assume the pandemic began earlier or later than March 2020, and report actual-minus-predicted overdose mortality per 100,000 people, by placebo-onset month, over the placebo pandemic period (from the placebo-onset month through March 2021).Vertical bars indicate 95% CIs. The left-hand figures show results for all drug overdoses; the right-hand figures show results for synthetic-opioid-related overdoses. Panel A uses the 2015 model, while Panel B uses the 2017 model. Each point on the graph represents the average difference between actual and predicted values of overdose deaths during the placebo-pandemic period, with prediction based on data from the pre-placebo-pandemic period.

With both the 2015 model and the 2017 model, and with both outcomes, the actual-minus predicted values for all overdose deaths do not change around March 2020, and generally become negative after the pandemic onset (opposite from predicted). These patterns are consistent with pre-pandemic trends driving higher pandemic-period mortality. They do not provide evidence that pandemic onset played a large role in mortality trends.

**Appendix Table 1- Approximate Numbers of Drug Overdose Deaths in San Francisco from Appa et al. (2021)**

**Note:** Numbers are approximate numbers drawn from the figure in Appa et al (2021) paper. This paper does not report exact numbers.

| **Year** | **2017** | **2018** | **2019** | **2020** |
| --- | --- | --- | --- | --- |
| January | 17 | 17 | 30 | 39 |
| February | 18 | 22 | 39 | 42 |
| March | 20 | 18 | 24 | 50 |
| April | 18 | 10 | 22 | 48 |
| May | 19 | 12 | 20 | 68 |
| June | 18 | 20 | 39 | 70 |
| July | 18 | 22 | 39 | 70 |
| August | 21 | 18 | 50 | 70 |
| September | 20 | 30 | 45 | 57 |
| October | 20 | 20 | 38 | 60 |
| November | 18 | 30 | 49 | 60 |
| December | 15 | 22 | 41 |  |
| Total | 222 | 241 | 436 | 634 |

**Appendix Figure 1- Placebo Estimates of Actual-minus-Predicted Drug Overdose Mortality Assuming Pandemic Started at Different Times**

National actual versus predicted drug overdose deaths per 100,000 persons between placebo pandemic-onset month and March 2021, assuming pandemic began in indicated placebo month from September 2019 through September 2020. Predicted values use 4th order polynomial, applied to data from 2015 (Panel A) or 2017 (Panel B) through month prior to assumed pandemic onset month. In each panel, left-hand figure shows results for all overdose deaths, and right-hand figure shows results for synthetic-opioid-related deaths. Vertical dashed line between February and March 2020 indicates start of pandemic period. Small vertical bars show 95% confidence intervals (CIs).

**Replacement figures:**

**Panel A: Year 2015 as Starting Point**

** Panel B: Year 2017 as Starting Point**

**Appendix Figure 1- Monthly Ratios of ED Visits for National Staffing Company During the COVID-19 Pandemic with Confidence Intervals**

Ratios for all visits, opioid overdose visits, and all other overdose visits for January 2017 through October 2021 using data from 181 EDs in 18 states staffed by a national emergency department staffing company. Ratios for 2020-2021 are to same month in 2019. We use standard errors clustered on ED and hospital weights based on 2019 volume to calculate 95% confidence intervals (CI).

**Appendix Figure 2- Monthly Ratios of ED Visits for National Staffing Company During the COVID-19 Pandemic For Balanced Panel**

Ratios for all visits, opioid overdose visits, and all other overdose visits for January 2017 through October 2021 using data from 37 Eds staffed by a national emergency department staffing company consistently from January 2016 through October 2021. Ratios for 2020-2021 are to same month in 2019. We use standard errors clustered on ED and hospital weights based on 2019 volume to calculate 95% confidence intervals (CI).

References

1. Appa A, Rodda LN, Cawley C, et al. Drug Overdose Deaths before and after Shelter-in-Place Orders during the COVID-19 Pandemic in San Francisco. *JAMA Netw Open*. 2021;4(5). doi:10.1001/jamanetworkopen.2021.10452

2. Fentanyl, heroin overdoses in San Francisco more than doubled in 2019. Accessed October 23, 2022. https://www.sfchronicle.com/bayarea/article/Fentanyl-heroin-overdoses-in-San-Francisco-more-14993628.php

3. Mason M, Arukumar P, Feinglass J. The Pandemic Stay-at-Home Order and Opioid-Involved Overdose Fatalities. *JAMA - Journal of the American Medical Association*. Published online 2021. doi:10.1001/jama.2021.6700

4. Currie JM, Schnell MK, Schwandt H, Zhang J. Trends in Drug Overdose Mortality in Ohio During the First 7 Months of the COVID-19 Pandemic. *JAMA Netw Open*. 2021;4(4):e217112-e217112. doi:10.1001/JAMANETWORKOPEN.2021.7112

5. Kelley MA, Lucas J, Stewart E, Goldman D, Doctor JN. Opioid-related deaths before and after COVID-19 stay-at-home orders in Los Angeles County. *Drug Alcohol Depend*. 2021;228:109028. doi:10.1016/J.DRUGALCDEP.2021.109028

6. Faust JS, Du C, Mayes KD, et al. Mortality From Drug Overdoses, Homicides, Unintentional Injuries, Motor Vehicle Crashes, and Suicides During the Pandemic, March-August 2020. *JAMA*. Published online May 21, 2021. doi:10.1001/jama.2021.8012

7. Soares WE, Melnick ER, Nath B, et al. Emergency Department Visits for Nonfatal Opioid Overdose During the COVID-19 Pandemic Across Six US Health Care Systems. *Ann Emerg Med*. Published online March 19, 2021. doi:10.1016/j.annemergmed.2021.03.013

8. Holland KM, Jones C, Vivolo-Kantor AM, et al. Trends in US Emergency Department Visits for Mental Health, Overdose, and Violence Outcomes Before and During the COVID-19 Pandemic Multimedia Supplemental content. *JAMA Psychiatry*. 2021;78(4):372-379. doi:10.1001/jamapsychiatry.2020.4402

9. Ochalek TA, Cumpston KL, Wills BK, Gal TS, Moeller FG. Nonfatal Opioid Overdoses at an Urban Emergency Department During the COVID-19 Pandemic. *JAMA*. 2020;324(16):1673-1674. doi:10.1001/JAMA.2020.17477

10. Slavova S, Rock P, Bush HM, Quesinberry D, Walsh SL. Signal of increased opioid overdose during COVID-19 from emergency medical services data. *Drug Alcohol Depend*. 2020;214:108176. doi:10.1016/J.DRUGALCDEP.2020.108176

11. Rodda LN, West KL, LeSaint KT. Opioid Overdose–Related Emergency Department Visits and Accidental Deaths during the COVID-19 Pandemic. *Journal of Urban Health*. 2020;97(6):808-813. doi:10.1007/S11524-020-00486-Y/FIGURES/3

12. Pines JM, Zocchi MS, Black BS, et al. How emergency department visits for substance use disorders have evolved during the early COVID-19 pandemic. *J Subst Abuse Treat*. 2021;129:108391. doi:10.1016/j.jsat.2021.108391
