# Supplementary material for: Estimating the uncertain effect of the COVID pandemic on drug overdoses
Source: PLoS One. 2023 Aug 10;18(8):e0281227. doi: 10.1371/journal.pone.0281227 (PMC10414597; doi:10.1371/journal.pone.0281227)
Supplement: Attachment — Submitted filename: Response to reviewers.docx [file pone.0281227.s005.docx]

Dear Professor Alfano,

Thank you for consideration of our paper, “Estimating The Uncertain Effect of the COVID Pandemic on Drug Overdoses.” Below, we have responded to the editor’s and reviewers’ comments.

PONE-D-23-01497. Estimating The Uncertain Effect of the COVID Pandemic on Drug Overdoses

Dear Dr. Moghtaderi,

Thank you for submitting your manuscript to PLOS ONE. After careful consideration, we feel that it has merit but does not fully meet PLOS ONE’s publication criteria as it currently stands. Therefore, we invite you to submit a revised version of the manuscript that addresses the points raised during the review process.

Please address the comments of reviewer 2, with special attention to ecological fallacy and the composition of the sample.

**Reply**. We respond to the referees below.

*Ecological fallacy*. Neither referee mentions an ecological fallacy issue, and we do not believe our study has this problem. This term is usually applies to situations where one infers individual-level characteristics from group results. We do not do this.

*Sample composition*. We rely partly on CDC data and partly on data from US Acute Care Solutions. We reviewed what we said about each data source, and revised for clarity. For overdose deaths, the national data is complete. For national emergency department (ED) visit data, the CDC draws on EDs from 42 states that submit data to it. The sample should be reasonably representative of larger EDs, but we don’t want to claim that because the CDC does not disclose which EDs participate. For both of our sources for ED visit data (CDC and the ED staffing company), we added information about visit counts to the text.

Please submit your revised manuscript by Apr 26 2023 11:59PM. If you will need more time than this to complete your revisions, please reply to this message or contact the journal office at [plosone@plos.org](mailto:plosone@plos.org). When you're ready to submit your revision, log on to <https://www.editorialmanager.com/pone/> and select the 'Submissions Needing Revision' folder to locate your manuscript file.

Please include the following items when submitting your revised manuscript:

- A rebuttal letter that responds to each point raised by the academic editor and reviewer(s). You should upload this letter as a separate file labeled 'Response to Reviewers'.
- A marked-up copy of your manuscript that highlights changes made to the original version. You should upload this as a separate file labeled 'Revised Manuscript with Track Changes'.
- An unmarked version of your revised paper without tracked changes. You should upload this as a separate file labeled 'Manuscript'.
- **Reply**. Done.

If you would like to make changes to your financial disclosure, please include your updated statement in your cover letter. Guidelines for resubmitting your figure files are available below the reviewer comments at the end of this letter.

If applicable, we recommend that you deposit your laboratory protocols in [protocols.io](https://urldefense.com/v3/__http:/protocols.io__;!!Dq0X2DkFhyF93HkjWTBQKhk!WQ7BYse28bIB7D_kZMJ9IUxqoEGIh5cY56LNkX9hZ9q6aRvR8OOQm4kKzcudT7ZnXl-Wmx5t33vwC50GkbEV-9502tc4rN3TAEbJPg$) to enhance the reproducibility of your results. Protocols.io assigns your protocol its own identifier (DOI) so that it can be cited independently in the future. For instructions see: [https://journals.plos.org/plosone/s/submission-guidelines#loc-laboratory-protocols](https://urldefense.com/v3/__https:/journals.plos.org/plosone/s/submission-guidelines*loc-laboratory-protocols__;Iw!!Dq0X2DkFhyF93HkjWTBQKhk!WQ7BYse28bIB7D_kZMJ9IUxqoEGIh5cY56LNkX9hZ9q6aRvR8OOQm4kKzcudT7ZnXl-Wmx5t33vwC50GkbEV-9502tc4rN2qHSCF3Q$). Additionally, PLOS ONE offers an option for publishing peer-reviewed Lab Protocol articles, which describe protocols hosted on [protocols.io](https://urldefense.com/v3/__http:/protocols.io__;!!Dq0X2DkFhyF93HkjWTBQKhk!WQ7BYse28bIB7D_kZMJ9IUxqoEGIh5cY56LNkX9hZ9q6aRvR8OOQm4kKzcudT7ZnXl-Wmx5t33vwC50GkbEV-9502tc4rN3TAEbJPg$). Read more information on sharing protocols at [https://plos.org/protocols?utm_medium=editorial-email&utm_source=authorletters&utm_campaign=protocols](https://urldefense.com/v3/__https:/plos.org/protocols?utm_medium=editorial-email&utm_source=authorletters&utm_campaign=protocols__;!!Dq0X2DkFhyF93HkjWTBQKhk!WQ7BYse28bIB7D_kZMJ9IUxqoEGIh5cY56LNkX9hZ9q6aRvR8OOQm4kKzcudT7ZnXl-Wmx5t33vwC50GkbEV-9502tc4rN2gTTFnQw$).

We look forward to receiving your revised manuscript.

Kind regards,

Vincenzo Alfano
Academic Editor
PLOS ONE

Journal Requirements:

When submitting your revision, we need you to address these additional requirements.

1. Please ensure that your manuscript meets PLOS ONE's style requirements, including those for file naming. The PLOS ONE style templates can be found at

[https://journals.plos.org/plosone/s/file?id=wjVg/PLOSOne_formatting_sample_main_body.pdf](https://urldefense.com/v3/__https:/journals.plos.org/plosone/s/file?id=wjVg*PLOSOne_formatting_sample_main_body.pdf__;Lw!!Dq0X2DkFhyF93HkjWTBQKhk!WQ7BYse28bIB7D_kZMJ9IUxqoEGIh5cY56LNkX9hZ9q6aRvR8OOQm4kKzcudT7ZnXl-Wmx5t33vwC50GkbEV-9502tc4rN3h4u2hbw$) and

[https://journals.plos.org/plosone/s/file?id=ba62/PLOSOne_formatting_sample_title_authors_affiliations.pdf](https://urldefense.com/v3/__https:/journals.plos.org/plosone/s/file?id=ba62*PLOSOne_formatting_sample_title_authors_affiliations.pdf__;Lw!!Dq0X2DkFhyF93HkjWTBQKhk!WQ7BYse28bIB7D_kZMJ9IUxqoEGIh5cY56LNkX9hZ9q6aRvR8OOQm4kKzcudT7ZnXl-Wmx5t33vwC50GkbEV-9502tc4rN0WbYxkyw$)

2. In your Data Availability statement, you have not specified where the minimal data set underlying the results described in your manuscript can be found. PLOS defines a study's minimal data set as the underlying data used to reach the conclusions drawn in the manuscript and any additional data required to replicate the reported study findings in their entirety. All PLOS journals require that the minimal data set be made fully available. For more information about our data policy, please see [http://journals.plos.org/plosone/s/data-availability](https://urldefense.com/v3/__http:/journals.plos.org/plosone/s/data-availability__;!!Dq0X2DkFhyF93HkjWTBQKhk!WQ7BYse28bIB7D_kZMJ9IUxqoEGIh5cY56LNkX9hZ9q6aRvR8OOQm4kKzcudT7ZnXl-Wmx5t33vwC50GkbEV-9502tc4rN205Krt3w$).

Upon re-submitting your revised manuscript, please upload your study’s minimal underlying data set as either Supporting Information files or to a stable, public repository and include the relevant URLs, DOIs, or accession numbers within your revised cover letter. For a list of acceptable repositories, please see [http://journals.plos.org/plosone/s/data-availability#loc-recommended-repositories](https://urldefense.com/v3/__http:/journals.plos.org/plosone/s/data-availability*loc-recommended-repositories__;Iw!!Dq0X2DkFhyF93HkjWTBQKhk!WQ7BYse28bIB7D_kZMJ9IUxqoEGIh5cY56LNkX9hZ9q6aRvR8OOQm4kKzcudT7ZnXl-Wmx5t33vwC50GkbEV-9502tc4rN1Ad9NQvg$). Any potentially identifying patient information must be fully anonymized.

Important: If there are ethical or legal restrictions to sharing your data publicly, please explain these restrictions in detail. Please see our guidelines for more information on what we consider unacceptable restrictions to publicly sharing data: [http://journals.plos.org/plosone/s/data-availability#loc-unacceptable-data-access-restrictions](https://urldefense.com/v3/__http:/journals.plos.org/plosone/s/data-availability*loc-unacceptable-data-access-restrictions__;Iw!!Dq0X2DkFhyF93HkjWTBQKhk!WQ7BYse28bIB7D_kZMJ9IUxqoEGIh5cY56LNkX9hZ9q6aRvR8OOQm4kKzcudT7ZnXl-Wmx5t33vwC50GkbEV-9502tc4rN3eTPBBbA$). Note that it is not acceptable for the authors to be the sole named individuals responsible for ensuring data access.

We will update your Data Availability statement to reflect the information you provide in your cover letter.

3. We note that you have stated that you will provide repository information for your data at acceptance. Should your manuscript be accepted for publication, we will hold it until you provide the relevant accession numbers or DOIs necessary to access your data. If you wish to make changes to your Data Availability statement, please describe these changes in your cover letter and we will update your Data Availability statement to reflect the information you provide.

4. PLOS requires an ORCID iD for the corresponding author in Editorial Manager on papers submitted after December 6th, 2016. Please ensure that you have an ORCID iD and that it is validated in Editorial Manager. To do this, go to ‘Update my Information’ (in the upper left-hand corner of the main menu), and click on the Fetch/Validate link next to the ORCID field. This will take you to the ORCID site and allow you to create a new iD or authenticate a pre-existing iD in Editorial Manager. Please see the following video for instructions on linking an ORCID iD to your Editorial Manager account: [https://www.youtube.com/watch?v=_xcclfuvtxQ](https://urldefense.com/v3/__https:/www.youtube.com/watch?v=_xcclfuvtxQ__;!!Dq0X2DkFhyF93HkjWTBQKhk!WQ7BYse28bIB7D_kZMJ9IUxqoEGIh5cY56LNkX9hZ9q6aRvR8OOQm4kKzcudT7ZnXl-Wmx5t33vwC50GkbEV-9502tc4rN0wYBjU6Q$)

5. Please include captions for your Supporting Information files at the end of your manuscript, and update any in-text citations to match accordingly. Please see our Supporting Information guidelines for more information: [http://journals.plos.org/plosone/s/supporting-information](https://urldefense.com/v3/__http:/journals.plos.org/plosone/s/supporting-information__;!!Dq0X2DkFhyF93HkjWTBQKhk!WQ7BYse28bIB7D_kZMJ9IUxqoEGIh5cY56LNkX9hZ9q6aRvR8OOQm4kKzcudT7ZnXl-Wmx5t33vwC50GkbEV-9502tc4rN2TzQC7pg$).

- **Reply**. Done.

**Reviewer's Responses to Questions**

**Comments to the Author**

1. Is the manuscript technically sound, and do the data support the conclusions?

The manuscript must describe a technically sound piece of scientific research with data that supports the conclusions. Experiments must have been conducted rigorously, with appropriate controls, replication, and sample sizes. The conclusions must be drawn appropriately based on the data presented.

Reviewer #1: Partly

Reviewer #2: Yes

2. Has the statistical analysis been performed appropriately and rigorously?

Reviewer #1: Yes

Reviewer #2: Yes

3. Have the authors made all data underlying the findings in their manuscript fully available?

The [PLOS Data policy](https://urldefense.com/v3/__http:/www.plosone.org/static/policies.action*sharing__;Iw!!Dq0X2DkFhyF93HkjWTBQKhk!WQ7BYse28bIB7D_kZMJ9IUxqoEGIh5cY56LNkX9hZ9q6aRvR8OOQm4kKzcudT7ZnXl-Wmx5t33vwC50GkbEV-9502tc4rN1UFSNkrg$) requires authors to make all data underlying the findings described in their manuscript fully available without restriction, with rare exception (please refer to the Data Availability Statement in the manuscript PDF file). The data should be provided as part of the manuscript or its supporting information, or deposited to a public repository. For example, in addition to summary statistics, the data points behind means, medians and variance measures should be available. If there are restrictions on publicly sharing data—e.g. participant privacy or use of data from a third party—those must be specified.

Reviewer #1: No

Reviewer #2: Yes

4. Is the manuscript presented in an intelligible fashion and written in standard English?

PLOS ONE does not copyedit accepted manuscripts, so the language in submitted articles must be clear, correct, and unambiguous. Any typographical or grammatical errors should be corrected at revision, so please note any specific errors here.

Reviewer #1: No

Reviewer #2: Yes

5. Review Comments to the Author

Please use the space provided to explain your answers to the questions above. You may also include additional comments for the author, including concerns about dual publication, research ethics, or publication ethics. (Please upload your review as an attachment if it exceeds 20,000 characters)

**Reviewer #1:**

This manuscript examined if increases in overdose deaths and ED visits were associated to the COVID-19 pandemic, increased synthetic-opioid use or both. The study takes into account pre-pandemic estimations to determine the effect of COVID-19 on Opioid overdose deaths and ED visits, which is novel and accurate comparing previous studies. Nonetheless, the manuscript needs clarity, as at times redundant and is not concise, which dilutes the findings.

**Reply**. We reread the manuscript, and tried to eliminate redundancy.

For example, there paragraphs: “Figure 1, Panel A, presents 12-month rolling average rates for all drug-related overdose deaths, synthetic-opioid-related deaths, and other overdose deaths, per 100,000 population”, but this type of information pertains to the figure, and results should be concise and state the statistical significance of what is added in the tables/figures, which is lacking in this section.

**Reply**. We added additional explanation to the text of the implications of this figure. This figure reports national totals as reported by the CDC; there are no associated confidence intervals. The CDC does not report them and we cannot compute them from the data that the CDC provides. We added an explanation to the limitations section that confidence intervals around the CDC-reported data are not available.

Additionally, the authors tend to be speculative at times in their discussion, for example using words like (i.e. somewhat lower), but don't back up these statements (is it statistically significant?)

**Reply**. We compare predicted deaths from our models (based on pre-pandemic trends) at different points in time, to actual mortality data from the CDC. The CDC data does not provide confidence intervals, so our models do not have them either. Thus, terms like “somewhat lower” are the best we can do, in describing how the prediction compares to the actual data.

We tried to be clear that any comparison of actual to predicted mortality is speculative.

Finally, there are moments where the authors state causality, but acknowledge that there could be other potential factors (such as the pandemic) that could be contributing to the predicted trends. With that said, the authors should avoid any causal jargon (even though the statistical model considers counterfactuals) and focus their study on the predictions found.

**Reply**. We tried to avoid any causal claims. For example, the draft stated that” Our model is not causal. Thus, we can neither rule out nor rule in a causal effect of fentanyl access or the pandemic on overdose deaths.” We added a similar statement to the introduction, saying we do not have a control group and therefore cannot have a causal research design. We reviewed the draft and the words used and revised for clarity. We believe that readers will understand that we are not making causal claims.

**Reviewer #2:**

The authors provide an in-depth and thorough review of the literature in their introduction that is relevant to their research question, and they provide adequate rationale for their study at hand.

What was the definition of death or non-death opioid presentations. Was this data determined from diagnosis codes used for ED visits, was this a clinical diagnosis, post-mortem, utilizing confirmatory blood levels? May be worth specifying.

**Reply**. Deaths come from the CDC, which relies on the text fields included in death certificates. The National Center for Health Statistics, within the CDC, received these death certificates, and assigns ICD-10 codes for primary cause of death and secondary causes based on the text fields. How accurate the death certificates are, and what blood tests or other laboratory results they are based on, is not known, and may vary based on who fills out the death certificate and customary practice where the person died. We explain this in the revised draft.

For ED visits, counts are based on diagnosis codes assigned by the clinician (usually an ED physician) at the time of the visit. We revised the draft to add details on this dataset.

Adequate explanation of limitations provided throughout, which is appreciated.

**Reply**. We added a limitation with regard to the accuracy of death certificates.

I believe the author's conclusions to be sound, and not overstated. There are limitations to the data presented, as the author's suggest; however, they do not drive their conclusions too far.
